# The boundary of holistic processing in the appraisal of facial attractiveness

**DOI:** 10.1098/rsos.171616

**Published:** 2018-06-20

**Authors:** Chang Hong Liu, Wenfeng Chen

**Affiliations:** 1 Department of Psychology, Bournemouth University, Fern Barrow, Poole, Dorset BH12 5BB, UK; 2 Department of Psychology, Renmin University of China, Beijing 100872, People's Republic of China

**Keywords:** facial attractiveness, external features, internal features, holistic processing

## Abstract

Facial attractiveness is often studied on the basis of the internal facial features alone. This study investigated how this exclusion of the external features affects the perception of attractiveness. We studied the effects of two most commonly used methods of exclusion, where the shape of an occluding mask was defined by either the facial outline or an oval. Participants rated attractiveness of the same faces under these conditions. Results showed that faces were consistently rated more attractive when they were masked by an oval shape rather than by their outline (Experiment 1). Attractive faces were more strongly affected by this effect than were less attractive faces when participants were able to control the viewing time. However, unattractive faces benefited more from this effect when the same face stimuli were presented briefly for only 20 ms (Experiment 2). Further manipulation confirmed that the effect was mainly due to the occlusion of a larger area of the external features rather than the regular and symmetrical features of the oval shape (Experiment 3) or lacks contextual cues about the face boundary (Experiment 4). The effect was only relative to masked faces, with no advantage over unmasked faces (Experiment 5), and is likely a result of the interaction between the shape of a mask and the internal features of the face. This holistic effect in the appraisal of facial attractiveness is striking, because the oval shape of the mask is not a part of the face but is the edge of an occluding object.

## 1. Introduction

**Figure 1.**
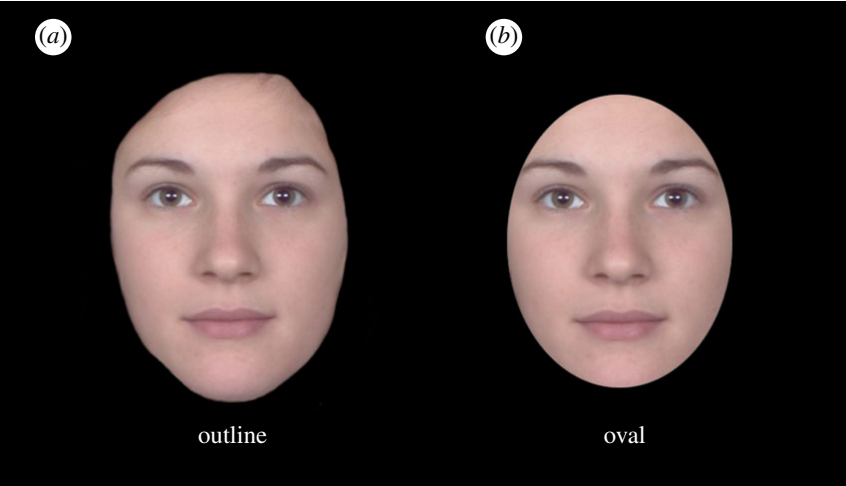
Two commonly used masking shapes to exclude the external features of a face. (*a*) Facial outline and (*b*) oval window. For copyright reasons, the example here was created from morphed identities.

Research on face perception routinely divides the face into internal and external features. Internal features consist of the eyes, nose and mouth, whereas external features include the hair, ears and facial outline. The distinction was first made in early studies of face recognition [1–3] and has been adopted in all areas of face research, including facial expression and facial attractiveness. Unlike scalp hair and paraphernalia, which can be easily altered, internal features are more stable aspects of a face. Research has focused on internal features by isolating them from the external region. The two most common methods for this control are shown in figure 1. The external features in both examples are masked, but to different extents. The first excludes the hair, whereas the second excludes a larger area, including the facial outline with an oval window. Although both are frequently used, research has rarely compared whether they produce the same effects. To understand whether these masking methods are comparable, we compared their effects on female facial beauty in this study. Owing to the holistic nature of face perception, the absence/presence of certain features could differentially affect the overall impression of a face. The shape of the jawline and hairline is hidden when an oval window is used as a mask. Hence these features cannot be used in attractiveness discrimination. Perhaps due to this consideration, numerous authors routinely include the shape of facial outline in their research on facial attractiveness (e.g. see masked face stimuli in [4–7]). However, it is unknown how the exclusion/inclusion of these features affects perceived attractiveness.

Occlusion is fairly common in natural images of faces. Occluding objects, such as headscarves and hijabs, can partially obscure the shape of the jawline and hairline. The hair and dark shadows can also hide portions of the forehead, eyebrows or cheekbones. Such naturally occurring partial occlusion seems to create little obstacles for evaluating facial beauty. The two masks in figure 1 could be seen as keyholes of different shapes that obscure different external regions of a face. Logically, because the occluding edge is not the same as the outline shape of a face, it should not affect the attractiveness of a face. This is particularly relevant to the oval mask in figure 1*b*, where the circular occluding edge is not a part of the face. The shape of the mask in figure 1*a*, however, coincides with that of the facial outline. Because of this, the shape is a relevant factor in the evaluation. If the shape of an occluding object does not affect the attractiveness of the face, the assessments of the two mask conditions should be equal because the rest of the two images are identical. However, an alternative account based on holistic processing would predict an interaction between the internal and external regions of face, which may be blind to the comprehension of edge ownership. That is, the edge around a face can be treated as a part of the face in an attractiveness judgement even though the observer can see whether it belongs to the face or an occluding object. If this is true, then even when it is seemingly impossible to assign an occluding edge to the face, the shape of the edge may automatically be included in holistic processing. A casual observation of the example in figure 1*a*,*b* appears to favour this hypothesis because it gives the impression that the face masked by an oval window looks more attractive. This simple observation was the starting point of this study. In a series of experiments, we measured the reliability and generality of this effect. In addition, we assessed whether this predicted advantage equally benefits faces of different levels of attractiveness.

There is ample evidence that an evaluation of facial attractiveness is holistic. In a composite paradigm, the attractiveness of the top half of the face is affected by the attractiveness of the bottom half when the two halves are aligned [8]. This means the two halves are automatically processed as a whole, even though the task requires judging only one half and ignoring the other. Although judging sexual appeal by a mate-relevant other sex could be based on specific facial features, assessing the aesthetic qualities of facial attractiveness relies heavily on holistic processing [9]. As a result of holistic processing, the same eye could appear larger or smaller, depending on its distance from the eyebrow [10]. Some key factors of facial attractiveness, such as averageness [11], symmetry [12] and sexual dimorphism [13], depend on a holistic integration of facial features. These factors are defined by statistical properties. The averageness hypothesis, for example, suggests that attractiveness depends on how close a face is to the statistical mean of a population of faces. Evidence for most of these factors has been created through the use of morphing techniques. For example, a face that has been averaged by morphing is commonly perceived as more attractive than the individual faces in the morph. However, research based on morphing techniques mainly reveals the contribution of internal features. This is because the technique requires clear corresponding points on the face images to be morphed. Although such points on the internal features are fairly easy to define, it can be difficult to find on the hair. Thus, the morphs created in most studies do not have aligned points for hair. Evidence based on morphing techniques for the contribution of symmetry and sexual dimorphism is also derived from internal features for the same reason. Our study is different from these studies because we looked into how an occluding shape may interact with the internal features in holistic processing.

The internal features of the face in this study remained constant in all experiments. Certain physical attributes of these features, such as large eyes, full lips, prominent cheekbones, and small nose and chin, are identified as the key contributors of facial beauty [14–17], personality and physical health [18]. However, external features also play a role in attractiveness judgements. This is evident from early infancy. Newborn infants pay attention to both internal and external features in their perception of attractiveness [19]. Adults also use both internal and external regions in attractiveness judgements [20]. External features such as hair can have a clear influence on attractiveness [21–23]. The shape and outline of a face can signal the person's youth, fertility, femininity and attractiveness [24,25]. In addition, skin texture plays an important role [26,27]. However, few studies have investigated how internal and external features interact in holistic processing of facial beauty. A notable exception can be found in Saegusa *et al*. [21], whose participants rated the attractiveness of a face or hair while ignoring task-irrelevant hair or faces. The results showed that task-irrelevant hair affected rating of the face if the participants had not previously rated the hair. By contrast, a task-irrelevant face always affected the attractiveness rating of hair regardless of whether the face had been rated before. This demonstrates an automatic interaction between face and hair in holistic processing. We were also interested in the interaction between the internal and external features. However, an important difference in our study is that the outline of the external features could also be the edge of an occluding object. In other words, our study also investigated the potential involvement of a surrounding non-face object in holistic face processing. This goes beyond the traditional inquiry about internal–external feature interaction.

The main hypothesis of this study was that internal facial features alone cannot determine perceived facial beauty. Rather, it is also determined by how the external region of a face is concealed or revealed. Although masking or occlusion does not affect the underlying physical shape of the face, the occluding edge of a mask or object could be automatically integrated with the face in holistic face processing.

## 2. Experiment 1

The first experiment addressed a simple question: Does the shape of a mask affect perceived attractiveness? We compared the effects of two masks (shown in figure 1) on attractiveness rating. One mask used the shape of a facial outline, whereas the other followed an oval shape. To study whether the two masks have the same effects on attractive and unattractive faces, we divided our stimuli into attractive and unattractive faces based on a prior rating of the original images.

Whether attractive and unattractive faces are equally affected by masking manipulation should depend on the extent to which external features or the shape of mask contributes equally at different levels of attractiveness. If, relative to attractive faces, external features account more for the perceived unattractiveness, then hiding these features with a mask should create a larger positive effect for unattractive faces than for attractive ones.

### 2.1. Method

#### 2.1.1. Participants

A total of 42 undergraduate students (30 females) aged between 20 and 30 years, *Mdn* = 21.5, participated in this experiment. All had normal or corrected-to-normal vision. The study was conducted in accordance with the American Psychological Association's guidelines on the treatment of human participants and was approved by the local ethics committee.

#### 2.1.2. Materials

The face database was obtained from the University of St Andrews. It has 702 frontal-view Caucasian faces pre-rated for facial attractiveness. Only the version of these faces masked by their facial outline was given a rating. The ratings were based on 19 raters on a 7-point scale. We only used female faces. To determine whether the masking manipulation in this study affects attractive and unattractive faces to the same degree, we selected the 24 most attractive and 24 least attractive female faces as our stimuli. The mean ratings for the two groups of faces were 4.11 and 2.23, respectively. These were significantly different from each other (*p* < 0.001).

Face width was normalized to 400 pixels, which subtended 16.6° of the visual angle. Each face was edited with Photoshop to create two versions. This resulted in a total of 96 face images (48 face identities × 2). The two versions were created via two mask shapes, as illustrated by the examples in figure 1. The ‘outline’ mask, which existed in the original face database, occluded the external features beyond the facial outline of the face. The ‘oval’ mask, in contrast, was a predefined oval window that occluded a greater area of external features, including the jawline and the hairline. The ratio of oval width to oval height was 1 : 1.3. It was adjusted to fit for the size of the face.

#### 2.1.3. Design and procedure

There were two independent variables. The first was Mask Shape (facial outline versus oval), and the second was Level of Attractiveness (attractive versus unattractive). The dependent variable was the attractiveness rating scores on a 7-point scale.

Participants were tested individually. Instructions were given on a computer screen. The face stimuli were displayed one at a time in the centre of the screen. A 1–7 scale was shown at the bottom of the screen. Participants were instructed to rate the attractiveness of the face presented using the scale, where 1 represented very unattractive and 7 represented very attractive. Participants used a computer mouse to click on the chosen point in the scale. The two masking conditions of each face identity were shown in separate blocks, which were separated by a 1 min break. Each face was randomly assigned to the first or the second block, such that the two conditions were intermixed in each block. The 48 face identities were randomized for each participant in the first block of trials. Thus, the attractive and unattractive faces were intermixed in each block. The face identities in the second block followed the same order of presentation as those in the first block such that the two versions of a face identity were maximally separated from each other in the experiment. This was done to minimize the potential influence of prior memory of the rating given to one version of a face and to encourage a response based on perceived attractiveness of the specific version rather than the memory of a previously given response.

### 2.2. Results

**Figure 2.**
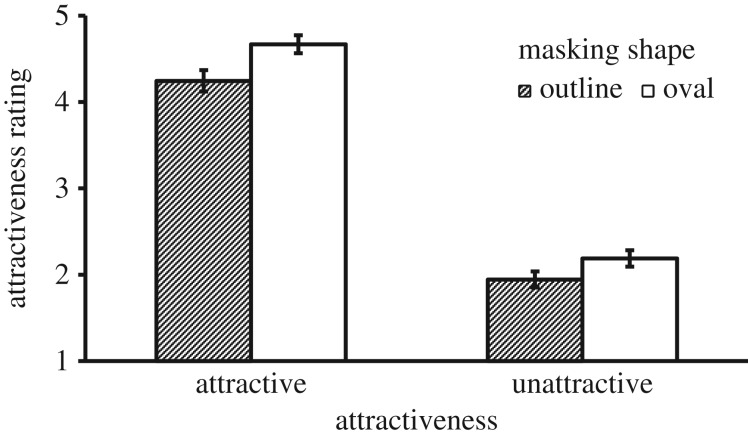
Mean attractiveness rating as a function of Masking Shape and Attractiveness in Experiment 1. Error bars represent 1 s.e. of the means.

There was a significant main effect of Mask Shape, *F*_1, 41_ = 56.68, *p* < 0.001, $\eta_{p}^{2}=0.580$, where the oval shape resulted in a higher attractiveness rating than the outline shape. There was also a main effect of Attractiveness, *F*_1, 41_ = 770.11, *p* < 0.001, $\eta_{p}^{2}=0.949$, where attractive faces were rated more attractive than unattractive ones. These main effects were qualified by a significant interaction, *F*_1, 41_ = 6.24, *p* = 0.017, $\eta_{p}^{2}=0.132$. The interaction effect can be seen in figure 2, which shows a greater difference between the results obtained for oval and outline shapes for attractive faces. To confirm this, we calculated the difference between ratings of oval and outline conditions for each participant, and then compared the two attractiveness conditions in a paired *t*-test. This showed that the attractive faces received a greater benefit from the oval mask than unattractive faces, *t*_41_ = 2.50, *p* = 0.017, Cohen's *d* = 0.78.

To assess whether masking affected all faces in a similar way or differently for different face identities, we performed an item-based rank correlation (Spearman's *ρ*) analysis. This produced fairly high correlations between the oval and outline mask conditions, *ρ* = 0.612 and 0.814 (*p*'s ≤ 0.001) for attractive and unattractive faces, respectively, indicating that masking affects all faces in a similar way.

### 2.3. Discussion

There were two notable findings in this experiment. First, the oval shape created a higher attractiveness rating for both attractive and unattractive faces. Second, this effect was stronger for attractive than for unattractive faces.

Although the advantage of the oval mask over the outline mask was expected, the finding that the effect was stronger for the more attractive faces was surprising. The interaction effect means that the gap between attractive and unattractive faces was greater when more external features of these faces were masked. Because the oval mask removed any difference between the facial outlines of attractive and unattractive faces, the perceived gap of attractiveness between these faces must have been mainly due to the differences in the internal face region. However, it should be noted that the exclusion of the outline could hide the facial adiposity or masculinity in these female faces, which would make them appear more attractive. The result also means that when more external features were present in the outline condition, these internal differences were less evident to the observers, whose attention might also be directed to the overall shape of a face.

It is possible that facial attractiveness judgements rely on different spatial scales for external and internal features. Discrimination of attractiveness among the facial outline may use more low spatial frequency information, whereas discrimination among the oval version may rely relatively more on high spatial frequency information. It is known that low-frequency information is processed more quickly, which may contribute to the attractiveness judgement after short exposure to the face [28,29]. There is also evidence that low-frequency information is important for the early stage of face processing, whereas high spatial frequency information is processed at a later stage of visual cognition [30,31]. The results in this experiment were likely to reflect a more advanced stage of processing because participants were given unlimited viewing time. However, past research has demonstrated that attractiveness appraisal can be made when images were exposed for one-tenth of a second or even less than 20 ms [32–34]. Moreover, unlike the eyes, which contribute to attractiveness judgements at a wide range of exposure duration, certain internal features such as nose and mouth can contribute more to facial attractiveness with an increasing level of exposure time [34]. When exposure time is increased, all faces tend to be perceived as less attractive [33,34]. This could be due to finer discrimination of imperfection detected at a late processing stage. However, it is not yet known whether this effect is associated with processing of fine spatial details of internal features. It is also unknown whether external features play a different role under a brief exposure. We thus investigated these questions in the next experiment.

## 3. Experiment 2

The purpose of this experiment was both to replicate the main findings of Experiment 1 and to test the hypothesis that external facial features (the outline face shape) play a more important role in determining the attractiveness of a face in a brief exposure. Internal features in a brief exposure condition should play a relatively minor role in judging attractiveness because these features are mainly carried by high spatial frequencies, which require a longer exposure time to process. We therefore expected a reduced difference between ratings of attractive and unattractive faces for the oval mask condition, where a judgement could only be based on internal features. By contrast, because external features are mainly carried by low spatial frequencies and processing of this information can occur quickly, we expected a relatively larger difference between attractive and unattractive faces for the outline version of the faces, where discrimination of attractiveness can rely more on external features.

### 3.1. Method

#### 3.1.1. Participants

A total of 45 university students (*Mdn* age 21 years, range 18–42 years, 32 females) participated in this experiment. All had normal or corrected-to-normal vision.

#### 3.1.2. Materials

The face stimuli were identical to those used in Experiment 1.

#### 3.1.3. Design and procedure

These were also identical to Experiment 1, except that exposure time was added as a new independent variable. Each face image was rated in two exposure conditions, once after a 20 ms presentation and once without a time limit, as in Experiment 1. The two conditions were tested in separate blocks. The order of the two blocks was counterbalanced across the participants.

### 3.2. Results

ANOVA showed significant main effects of Exposure Time, where the same faces presented in a brief exposure were rated more attractive than in a long exposure, *F*_1,41_ = 101.43, *p* < 0.001, $\eta_{p}^{2}=0.712$, Mask Shape, where faces with the oval mask were rated more attractive relative to the outline mask, *F*_1,41_ = 118.58, *p* < 0.001, $\eta_{p}^{2}=0.743$, and Attractiveness, *F*_1,41_ = 906.15, *p* < 0.001, $\eta_{p}^{2}=0.957$. These effects were qualified by a two-way interaction between Exposure Time and Mask Shape, *F*_1, 41_ = 12.37, *p* = 0.001, $\eta_{p}^{2}=0.232$, and a three-way interaction, *F*_1,41_ = 11.72, *p* = 0.001, $\eta_{p}^{2}=0.222$.

**Figure 3.**
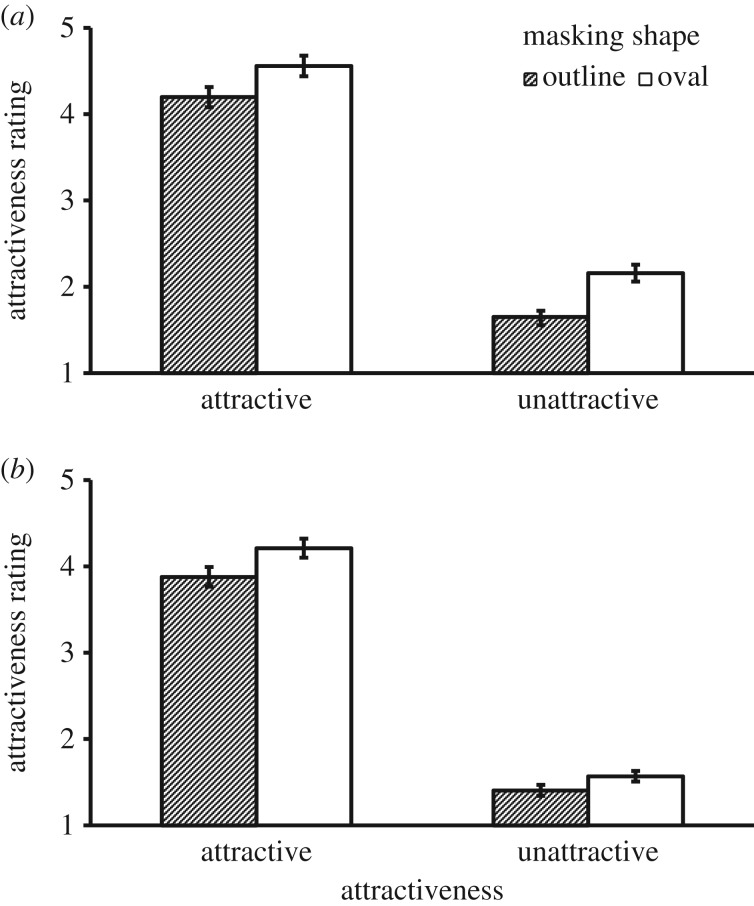
Mean attractiveness rating as a function of Masking Shape and Attractiveness in Experiment 2. (*a*) Brief exposure and (*b*) long exposure. Error bars represent 1 s.e. of the means.

To identify the source of the three-way interaction, we conducted simple effects analyses separately for the two exposure conditions. For the brief exposure condition, there was a significant main effect of Mask Shape, *F*_1, 41_ = 101.80, *p* < 0.001, $\eta_{p}^{2}=0.713$, where the oval mask shape created higher ratings. There was also a main effect of Attractiveness, *F*_1, 41_ = 708.16, *p* < 0.001, $\eta_{p}^{2}=0.945$, where faces that were pre-rated as being attractive were rated as more attractive. Furthermore, there was a Mask Shape × Attractiveness interaction, *F*_1, 41_ = 4.32, *p* = 0.044, $\eta_{p}^{2}=0.095$. However, the interaction was due to a different pattern of results from Experiment 1, which is illustrated in figure 3*a*. To compare the size of the effects for attractive and unattractive faces, we analysed the two-way interaction by computing the effect of Mask Shape followed a paired *t*-test. This showed that the attractive faces received less benefit from the oval shape than unattractive faces, *t*_41_ = −2.08, *p* = 0.044, Cohen's *d* = 0.65. This result was the reverse of the effect of the long exposure condition and the effect of Experiment 1. It showed that in a brief 20 ms exposure, masking by an oval window was more beneficial for unattractive faces.

Simple effects analyses for the long exposure condition also showed similar main effects of Mask Shape, *F*_1, 42_ = 40.22, *p* < 0.001, $\eta_{p}^{2}=0.489$, and Attractiveness, *F*_1, 42_ = 848.60, *p* < 0.001, $\eta_{p}^{2}=0.953$. There was also a Mask Shape × Attractiveness interaction, *F*_1, 42_ = 6.69, *p* = 0.013, $\eta_{p}^{2}=0.137$. This interaction is illustrated in figure 3*b*. To compare the size of the effects for attractive and unattractive faces, we again analysed the two-way interaction by computing the effect of Mask Shape followed by a paired *t*-test. This showed that the attractive faces received greater benefit from the oval shape than unattractive faces, *t*_42_ = 2.57, *p* = .014, Cohen's *d* = 0.80. The results replicated the findings in Experiment 1. However, we should note that although the overall pattern of results was consistent with Experiment 1, the overall mean ratings in the long exposure condition were notably lower in this experiment.

We also conducted simple effects analyses by separating the two mask conditions. For the outline mask condition, there were significant main effects of Exposure Time and Attractiveness, as revealed in the earlier analyses. The interaction between these factors was not significant. For the oval mask condition, however, apart from the same main effects, there was a significant interaction between these factors, *F*_1, 41_ = 6.89, *p* = 0.012, $\eta_{p}^{2}=0.144$. Although a longer exposure reduced the attractiveness of both attractive and unattractive faces, the degree of reduction was greater for unattractive faces than for attractive faces, *t*_41_ = −2.63, *p* = 0.012, Cohen's *d* = 0.82.

Finally, to assess how different levels of attractiveness were affected by these variables, we also conducted separate simple effects analyses for each attractiveness level. For the attractive faces, both main effects of Exposure Time and Mask Shape were as significant as the previous analyses. No interaction was found between the variables. For unattractive faces, however, apart from the same main effects, there was also a significant interaction, *F*_1, 41_ = 39.77, *p* < 0.001, $\eta_{p}^{2}=0.492$. This was due to a larger difference between the oval and outline mask conditions in the brief exposure relative to the long exposure, *t*_41_ = −6.31, *p* < 0.001, Cohen's *d* = 1.97.

As in Experiment 1, we also conducted an item-based rank correlation analysis to assess whether masking affected all faces in a similar way. The results showed significant correlations between oval and outline masks, *ρ* = 0.722 and 0.797 (*p*'s < 0.01), respectively, for attractive and unattractive faces in the long exposure conditions. In brief exposure conditions, the correlations were 0.646 and 0.721 (*p*'s < 0.01), respectively, for attractive and unattractive faces.

As the overall mean ratings in the long exposure condition appeared to be lower than the comparable exposure condition in Experiment 1, we also calculated the rank correlations of the faces across the conditions between the two experiments. The Spearman *ρ* is 0.91 for oval mask, and 0.96 for outline mask. The inter-rater reliability (intraclass correlation coefficient) for unattractive faces is 0.983 for the oval mask condition and 0.978 for the outline mask condition. These analyses suggest that the results across the two conditions were fairly consistent.

### 3.3. Discussion

Replicating Experiment 1, the oval mask condition again received a higher attractiveness rating relative to the outline condition in this experiment. Although the effect was found in both exposure conditions, the results confirm the hypothesis that attractive faces benefit more from the effect when face stimuli were shown for a longer duration, whereas unattractive faces receive more benefit from the effect when face stimuli were shown for a brief duration. The experiment also replicated a previous finding that a longer exposure time decreases the overall attractiveness rating [33,34]. Attractiveness judgements may depend on collecting evidence from both low and high spatial frequencies. Judgements in a brief exposure are likely to rely more on low spatial frequency information such as the facial outline. A longer exposure could reveal more detailed features and imperfections supported by high-frequency information. This could explain the decrease of initially perceived attractiveness.

This experiment further extended prior findings by showing that the effect of exposure time may have different strengths depending on the level of attractiveness and the way the face stimuli are presented. In the oval mask condition, the effect of longer exposure was greater for unattractive faces than for attractive ones. This effect suggests that a full identification of unattractive features may rely more on accessing the high spatial frequency information. The results also showed that the strength of the oval mask advantage in the two exposure conditions was the same for the attractive faces and that the strength of this advantage for the unattractive faces was greater in the brief exposure relative to the long exposure.

Although the masking shape effects in the first two experiments could be due to greater reduction of external features in the masked faces, there is also an alternative explanation. Using an oval shape not only concealed more external features but also introduced a highly regular and symmetrical shape. Hence the effect found in Experiments 1 and 2 could be due to the advantage of a more regular and symmetrical shape. To determine whether the oval shape alone could explain the effect, we conducted Experiment 3.

## 4. Experiment 3

The determine whether the oval mask effect in Experiments 1 and 2 was due to the reduced contribution from the external features or a more regular and symmetrical shape, we attempted to replicate Experiment 1 but added a third masking condition. In the new condition, roughly the same area was masked, as in the oval mask condition, but with an irregular shape that was an identical irregular shape, as in the facial outline condition. In other words, we reduced the aperture size of the mask. If the regular and symmetrical feature alone could explain the oval mask effect, there should be no difference in the ratings of the two outline conditions. If there were a clear advantage in the reduced outline condition but the difference between this condition and the oval mask condition were minimal, it would suggest that the oval mask effect was mainly due to a reduction of external features.

### 4.1. Method

#### 4.1.1. Participants

Forty-five university students (*Mdn* age 21 years, range 18–42 years, 31 females) participated in the experiment.

#### 4.1.2. Materials

**Figure 4.**
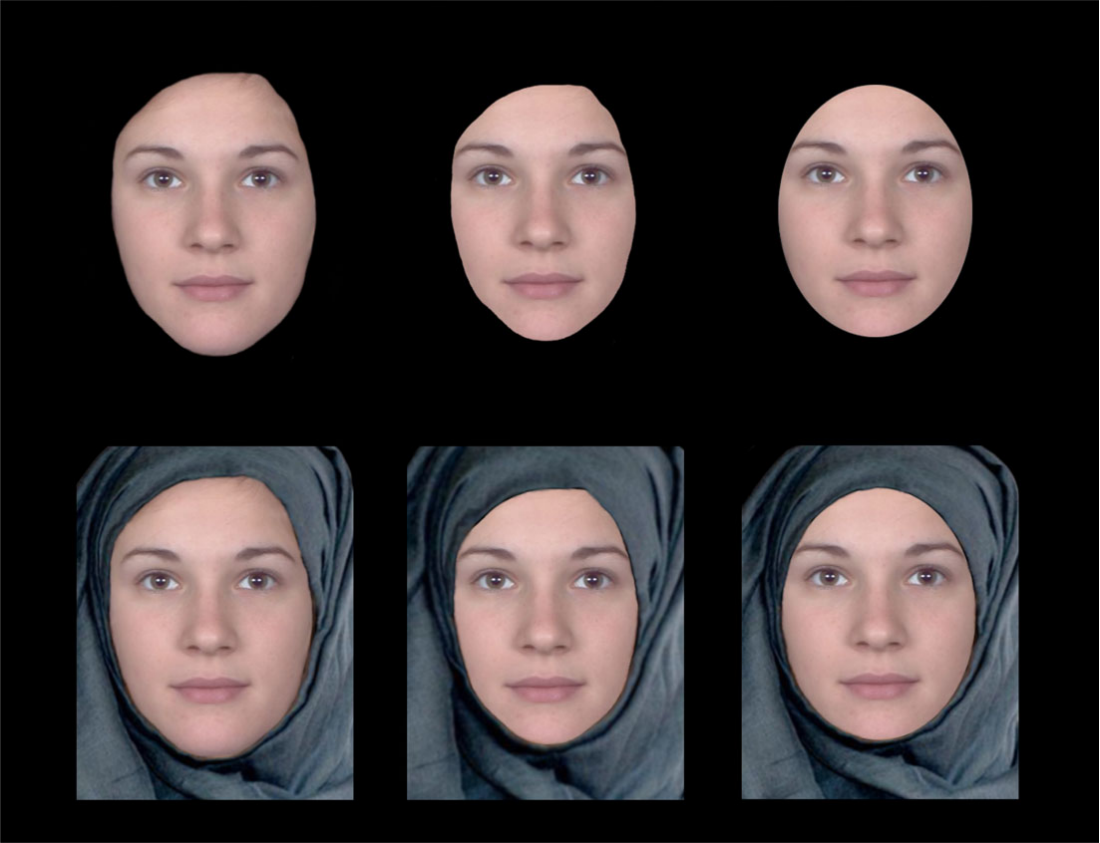
Examples of the masking conditions in Experiments 3 (top row) and 4 (bottom row). The three conditions are ‘outline’ (left column), ‘reduced outline’ (middle column) and ‘oval’ (right column). For copyright reasons, the example was created from morphed identities.

These were identical to Experiments 1 and 2, except a new mask version was added for each face identity. The shape of the new mask was identical to the facial outline mask, except the aperture size was reduced by 15% to obscure approximately the same external face area as the oval-shaped mask. In contrast to the oval mask, however, it had an irregular shape. Figure 4 illustrates the new version among the old versions of an example face.

#### 4.1.3. Design and procedure

This was a within-participant design. The two factors were Mask Shape (outline, reduced outline and oval) and Attractiveness (attractive versus unattractive).

The procedure was essentially the same as in Experiment 1, except a block of 48 trials was added to accommodate the additional, reduced outline condition. As in Experiment 1, each trial within one block showed a unique face, and a different version of a face was presented in a different block. The randomization procedure was also the same as in Experiment 1, hence all conditions were mixed in each block. The 48 face identities with three masking conditions amounted to a total of 144 trials. All other aspects of the procedure and task were identical to those in Experiment 1.

### 4.2. Results

**Figure 5.**
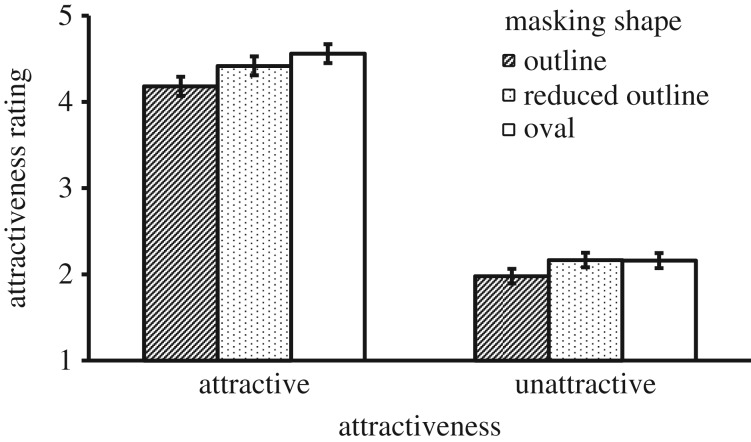
Mean attractiveness rating as a function of Mask Shape and Attractiveness in Experiment 3. Error bars represent 1 s.e. of the means.

The results of this experiment are shown in figure 5. ANOVA showed significant main effects of Mask Shape, *F*_2, 88_ = 40.79, *p* < 0.001, $\eta_{p}^{2}=0.481$, and Attractiveness, *F*_1, 44_ = 566.06, *p* < 0.001, $\eta_{p}^{2}=0.928$. These effects were also qualified by a significant interaction, *F*_2, 88_ = 5.09, *p* = 0.008, $\eta_{p}^{2}=0.104$. Simple effects analyses showed a significant main effect of Mask Shape for attractive faces, *F*_2, 88_ = 24.18, *p* < 0.001, $\eta_{p}^{2}=0.355$. Pairwise comparisons with Bonferroni correction showed that the oval mask condition produced a higher attractiveness rating than the reduced outline mask, *p* = 0.038, Cohen's *d* = 0.79, which in turn produced a higher rating than the outline mask condition, *p* < 0.001, Cohen's *d* = 1.25. The main effect of Mask Shape was also significant for unattractive faces, *F*_2, 88_ = 19.26, *p* < 0.001, $\eta_{p}^{2}=0.304$. Pairwise comparisons showed that both oval and irregular mask conditions produced a higher attractiveness rating than did the outline condition, *p's *< 0.001, Cohen's *d* = 1.73 and 1.41. The rating scores for the oval and irregular mask conditions were comparable, *p* = 1.00.

As previous experiments, we performed item-based rank correlation analyses to assess whether masking affected all faces in a similar way. The results showed high correlations between masking conditions. For the attractive faces, the correlations ranged from 0.651 to 0.913, *p*'s ≤ 0.001. For the unattractive faces, the correlations ranged from 0.753 to 0.804, *p*'s < 0.001.

### 4.3. Discussion

The results of this experiment show that the reduced outline condition had a clear advantage over the outline condition for both attractive and unattractive faces. The oval shape condition was also rated higher than the reduced outline condition for attractive faces. There was no difference in the perceived attractiveness of these two conditions for unattractive faces. The results suggest that the relative advantage of the oval mask found in Experiments 1 and 2 was mainly explained by a greater occlusion of the external region of the face. The regular and symmetrical outline of the oval shape may have played a role but may not be a major contributing factor, because there was no difference between the reduced outline and the oval conditions for unattractive faces. These results were consistent with some prior research, which showed that an explicit feature-based measurement of facial symmetry was not correlated with human judgements of facial attractiveness [35,36].

There was a fundamental difference between the three masked versions of faces. In the outline condition, the outline was the actual shape of the face, but in the other two conditions, the outline was more likely to be seen as the occluding edge of the mask rather than a part of the face. However, is it possible that due to the lack of context in these images, the contour that belongs to the mask was interpreted as belonging to the face? For the outline version, it was a correct interpretation because the mask shape overlapped with the face shape. However, in the other two conditions, could the shape of the mask be mistakenly interpreted as the face shape? If the faces were shown in a less ambiguous context, such that the mask shape could be more readily interpreted as the edge of an occluding object, would the results in this experiment be different? We examined this possibility in the next experiment.

## 5. Experiment 4

If the effect of the mask in the previous experiments was due to a misinterpretation of outline ownership, can the effect be eradicated when the ambiguity is removed with a context? We tested this alternative explanation in this experiment. The design and task were exactly the same as in Experiment 3. The only difference was that we added a headscarf to each face image.

The effect of a headscarf on perception of facial attractiveness has been studied before. Mahmud & Swami [37] compared the attractiveness rating of faces with or without a hijab. They found that women wearing a hijab were rated less attractive than women without a hijab. The purpose of our study, however, was quite different from theirs. Our aim was not to determine whether wearing a headscarf could make a face more or less attractive than without the headscarf. All faces in this experiment wore a headscarf. Rather, our aim was to measure how different levels of masking by the headscarf affect perceived facial attractiveness.

### 5.1. Method

#### 5.1.1. Participants

Thirty university students (mean age 20.6 years, s.d. = 2.9 years, range 20–29 years, 27 females) participated. All had normal or corrected-to-normal vision.

#### 5.1.2. Materials

These were identical to Experiment 3, except a headscarf was digitally added to all face images with Photoshop using the Puppet Warp tool. We used the same headscarf in all face stimuli to avoid introducing extraneous variables. An illustration of this is given in figure 4 (second row).

#### 5.1.3. Design and procedure

These were identical to Experiment 3.

### 5.2. Results

**Figure 6.**
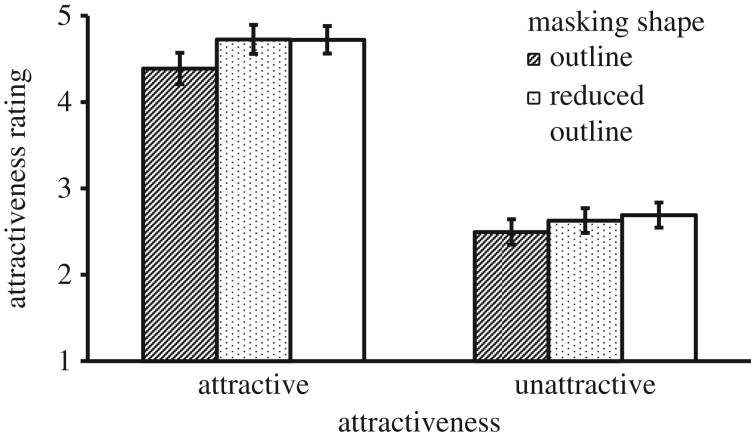
Mean attractiveness rating as a function of Masking Shape and Attractiveness in Experiment 4. Error bars represent 1 s.e. of the means.

Results are shown in figure 6. Similar to Experiment 3, there were significant main effects of Mask Shape, *F*_2, 58_ = 42.35, *p* < 0.001, $\eta_{p}^{2}=0.594$, and Attractiveness, *F*_1, 29_ = 277.22, *p* < 0.001, $\eta_{p}^{2}=0.905$. Moreover, these main effects were qualified by a significant interaction, *F*_2, 58_ = 6.40, *p* = 0.007, $\eta_{p}^{2}=0.181$. Simple effects analyses showed a significant main effect of attractive faces, *F*_2, 58_ = 28.12, *p* < 0.001, $\eta_{p}^{2}=0.492$. Paired comparisons with Bonferroni correction showed higher attractiveness ratings for oval and reduced outline masks relative to outline mask, *p*'s < 0.001, Cohen's *d* = 2.10 and 2.19, while the ratings for oval and reduced outline mask were the same (*p* = 1.00). The simple main effect of Mask Shape for unattractive faces was also significant, *F*_2, 58_ = 19.40, *p* < 0.001, $\eta_{p}^{2}=0.401$. Here, both oval and reduced outline mask conditions were rated as more attractive than the outline mask condition, *p*'s < 0.001 and = 0.002, Cohen's *d* = 2.33 and 1.45, respectively. The ratings for the oval and reduced outline conditions were comparable, *p* = 0.145.

We again conducted item-based rank correlation analysis to assess whether masking affected all faces in a similar way. For the attractive faces, the correlation between masking conditions ranged from 0.714 to 0.892, *p*'s < 0.001. For the unattractive faces, the correlation ranged from 0.778 to 0.827, *p*'s < 0.001.

### 5.3. Discussion

The pattern of results in the two experiments was consistent with Experiment 3. The only difference was that the oval mask advantage over the reduced outline mask condition for attractive faces failed to replicate in this experiment. This may mean that the small difference found between the conditions in Experiment 3 was not as robust as the other key findings. The overall pattern of the results in this experiment further confirms that the advantage of masking a greater area of external features relative to the more revealing outline mask was not due to a symmetrical or regular attribute of the oval mask or the lack of face context. A more plausible explanation is that mask shape was treated as a part of the face in holistic processing. We should note, however, that this does not mean the participants would not be able to distinguish mask shape from face shape if they were asked to do so. It only means that holistic processing may have included mask shape as a part of the face. This process is of course not accessible to conscious inspection.

The advantage of concealing a larger area of external features in the four experiments so far was demonstrated relative to the outline mask condition, where the jawline and hairline were fully visible. However, because the outline mask condition in the previous experiments did not include complete external features or context, it is difficult to ascertain whether the disadvantage of the outline mask relative to the other two mask conditions also extends to a no mask condition, in which full external features are shown. As appraisal of facial attractiveness is likely to be the result of holistic processing of the entire face, where external features interact with internal features, it is possible that evaluating a face through an outline mask is different from evaluating it without a mask. To find the answer, we included unmasked whole faces in the next experiment.

## 6. Experiment 5

Without comparing the rating of the masked faces to the original unmasked version, it would not be clear whether the outline mask version made a face less attractive or the oval version made the face more attractive relative to the unmasked version. This experiment aimed to address this question by including the original unmasked version of each face. To the best of our knowledge, no research has compared the attractiveness ratings of faces masked by both versions of external masks with the unmasked whole faces. A prior study by Santos & Young [20] did compare attractiveness judgements of faces masked by a circular window with the unmasked whole-face condition. They found that judgements of attractiveness for the whole-face condition were in higher agreement with the past judgements of the unmasked faces by different participants. Because they did not report which condition received the higher attractiveness rating, it is unclear whether the faces with a circular window mask were judged differently from those unmasked whole faces. We hoped to find an answer to this and the relative effect of the outline mask condition in the present experiment.

### 6.1. Method

#### 6.1.1. Participants

Thirty-seven university students (*Mdn* age 19 years, range 18–22 years, 33 females) participated in the experiment. All had normal or corrected-to-normal vision.

#### 6.1.2. Materials

**Figure 7.**
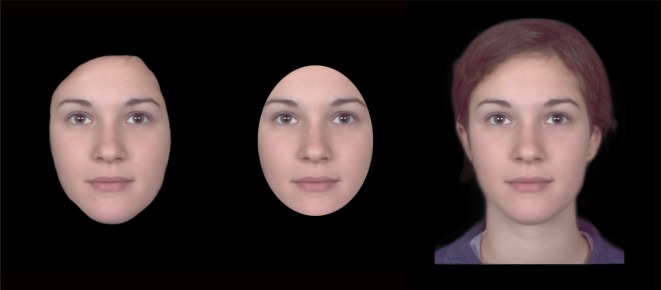
Three mask conditions in Experiment 5. From left to right: facial outline, oval and unmasked whole face. For copyright reasons, the example was created from morphed identities.

These were identical to Experiment 3, except the face images for the reduced outline condition were replaced with images with full external features. Figure 7 shows an example face in the three conditions.

#### 6.1.3. Design and procedure

Apart from replacing the reduced outline mask condition with the unmasked whole-face condition, these were identical to Experiment 3.

### 6.2. Results

**Figure 8.**
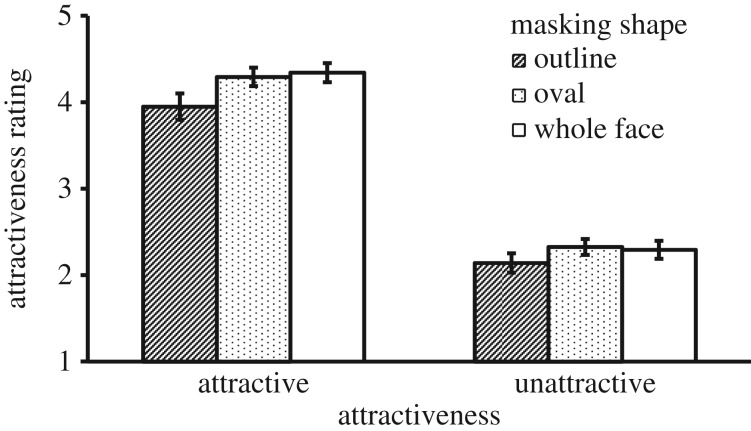
Mean attractiveness rating as a function of Masking Shape and Attractiveness in Experiment 5. Error bars represent 1 s.e. of the means.

Results are shown in figure 8. There were significant main effects of Mask Shape, *F*_2, 72_ = 8.48, *p* = 0.001, $\eta_{p}^{2}=0.191$, and Attractiveness, *F*_1, 36_ = 436.43, *p* < 0.001, $\eta_{p}^{2}=0.924$. The interaction between the two variables was also significant, *F*_2, 72_ = 5.24, *p* = 0.008, $\eta_{p}^{2}=0.127$. Simple effects analyses showed a significant effect of Mask Shape for attractive faces, *F*_2, 72_ = 8.93, *p* = 0.001, $\eta_{p}^{2}=0.199$, where ratings for the oval mask condition were higher than for the outline mask condition, *p* = 0.004, Cohen's *d* = 1.16, and ratings for the no mask, whole-face condition were also higher than the outline mask condition, *p* = 0.008, Cohen's *d* = 1.08. There was no difference between the ratings for the oval and whole-face conditions. There was also a significant effect of Mask Shape for unattractive faces, *F*_2, 72_ = 4.95, *p* = 0.011, $\eta_{p}^{2}=0.121$, where the rating for the oval mask condition was higher than that for the outline mask condition, *p* = 0.008, Cohen's *d* = 1.08. The ratings for the whole-face and outline mask conditions were comparable, *p* = 0.099. The ratings for the whole-face and oval mask conditions were also comparable, *p* = 1.000.

As in the previous experiments, we conducted item-based rank correlation analyses to assess whether masking affected all faces in a similar way. The results showed fairly high correlations between mask conditions. The correlation ranged from 0.721 to 0.769 for attractive faces, and 0.668 to 0.749 for unattractive faces, *p*'s ≤ 0.001.

The ratings of whole faces are comparable to the original rating from the database, for attractive faces, *M *= 4.25 versus 4.31, *t*_19_ = 0.49, *p* = 0.631; for unattractive faces, *M *= 2.22 versus 2.09, *t*_19_ = 1.71, *p* = 0.104.

Apart from the item-based correlation analyses, we also compared the same masking conditions across experiments to evaluate inter-rater reliability of attractiveness. This was done for the outline and oval masks because they were used across all five experiments in this study. The intraclass correlation coefficient was 0.989 for oval shape mask, and 0.989 for outline mask condition.

### 6.3. Discussion

The results in this experiment confirmed the previous finding that the oval mask condition created a higher attractiveness rating than the outline mask condition. This effect was again stronger for attractive faces than for unattractive faces. The novel finding in this experiment was that the oval mask condition had no advantage over the unmasked whole-face condition. This finding suggests that the oval mask advantage in the previous experiments was likely due to a reduced attractiveness suffered by the outline mask condition.

## 7. General discussion

We compared how two popular methods for masking varying amounts of external face features influence perceived facial attractiveness. All five experiments showed that masking a larger area of external features with an oval window resulted in a more attractive-looking face relative to masking external features, according to the shape of facial outline. Although the effect was found for both attractive and unattractive faces, the size of the effect was consistently stronger for attractive than for unattractive faces in these experiments, with the exception of a brief exposure condition (Experiment 2) when faces were shown for 20 ms rather than for an unlimited viewing time. After verifying these basic findings in Experiments 1 and 2, we showed in the subsequent experiments that the effects could not be easily explained by the symmetrical shape of the oval mask (Experiment 3) or the lack of face context (Experiment 4). Finally, by comparing the same masking conditions used in the first two experiments with a whole-face condition where no masking of external features was used, we were able to establish that the effect was due to a reduced attractiveness of the outline masking rather than increased attractiveness of the oval mask condition.

These findings have a number of theoretical implications. The first is related to the question of face boundary. Logically, when the attractiveness of a face is judged, the judgement should be about the face within its boundary, rather than being influenced by the shape of an adjacent object. However, our results appear to reject this. Indeed, the most striking aspect of our findings is that although the shape of the occluding object is not a shape of the face, it clearly affected the judgements of facial attractiveness. If participants were asked to judge the face shape, they would be unlikely to treat the oval shape as the boundary of a face. They should be able to see this more clearly in Experiment 4, where the oval-shaped outline was likely to be seen as belonging to the headscarf. However, they still perceived faces with the oval mask as being more attractive. This effect is equivalent to peeping at a face through keyholes of two different sizes, where one reveals only a small area of internal features and conceals more external features, and the other reveals greater external features and conceals only the part beyond the facial outline. Should the judgements of facial attractiveness be affected by the size of keyholes? Our results showed that they are.

This leads to another implication of our findings: perception of facial attractiveness could be a result of interaction between the internal region of the face and the shape of peripheral occluding objects. This means that the boundary of face processing does not stop at the face boundary. In other words, the edge of the occluding object could be a part of the holistic face processing even though it is not a part of the face. Past research has shown that makeup can alter a face's perceived attractiveness. However, this is usually achieved by modifying the appearance of certain facial features, such as eye-region contrast or apparent eye size (e.g. [10,38]). Our findings are quite different because we showed that the boundary of an occluding object that made no changes to a face could also influence perceived facial attractiveness. Future studies will need to delineate the boundary, the extent of vicinity and the conditions for the inclusion.

Identical internal features were used in our experiments, but changing the shape of the occluding edge with a mask consistently modulated perceived attractiveness. This demonstrates that internal features are influenced by the presence of external features. Occluding a larger area of external features appears to make the eyes and other internal features look larger relative to the occluding shape that followed a facial outline. This could account for the enhanced attractiveness, as neonate features such as large eyes and thick lips tend to make a face look more attractive [14–16]. Hence, the effect in our study could be the apparent size of the internal features when larger external features are masked. Curiously, however, the apparent size difference seems to vanish when faces with the oval mask are compared with their unmasked versions. This could explain why the effect disappears when the two conditions were compared in Experiment 5. Masking external features by the facial outline appears to make the face larger and wider compared with the unmasked whole-face version. If these casual observations were true, then the apparent size of internal features relative to the area bound by the facial outline could account for the effects observed in this study. The reduced attractiveness after scalp hair and other external features was masked by the facial outline relative to other conditions could be due to the apparent reduction in size of the internal features. These observations will require empirical evidence in future studies. Although the mechanisms behind this remain unknown, the absence of the features beyond the facial outline is most likely associated with reduced attractiveness. The missing features are clearly crucial for holistic face processing to produce a more favourable impression of a face size relative to the size of internal features.

Another consistent finding in our experiments was that attractive faces benefited more from extra masking when participants were given unlimited time to rate face stimuli. Unattractive faces may benefit more from the masking effect when face stimuli were briefly shown for 20 ms (Experiment 2). Internal features were likely to play a greater role in the effect of the longer exposure condition, whereas external features were likely to play a greater role in the brief exposure condition. In a brief exposure, the difference between attractive and unattractive faces could be more salient in the outline mask condition. This is because a judgement in this condition must rely more on the overall face shape, which is supported by coarse spatial information. A judgement cannot rely on this information when it is made unavailable in the oval mask condition. With a longer exposure, however, participants should be able to appraise attractiveness in this condition.

Our results demonstrate that methods used to mask or remove external features could alter perceived facial attractiveness in different ways. Because a face masked by its outline shape could reduce attractiveness relative to its unmasked version, and because this effect could be different for attractive and unattractive faces, it is necessary to exercise caution when attractiveness measured from stimuli with an outline mask is used to represent the attractiveness of the unmasked version. Although no difference was found between the perceived attractiveness of the oval mask and the unmasked version, it is important not to assume that the same mechanisms underpin the two conditions, as the outcome involved the holistic processing of different face regions.

Occlusion or masking, in reality, may have a similar effect as shown in our laboratory conditions. However, this will require further research. Although we used digitally manipulated headscarves to mimic different extents of occlusion, the technique may have inadvertently introduced elements that do not fully match the impression of headscarf shapes in reality. Furthermore, faces in reality could be partially occluded by many different objects and shapes. Our study supports the idea that facial beauty can be influenced by both intrinsic factors such as the shape of the features determined by biology and extraneous factors, such as hairstyle and occlusion. This suggests the possibility that even the way in which a headscarf is used to obscure different areas can significantly alter the impression of facial attractiveness.

Perhaps the most important message in this study is that holistic processing of facial attractiveness may extend to the shape of the occluding and adjacent object. Exactly how these elements interact with a face in a computational process remains an unresolved mystery.

## Data accessibility

Data are available at Dryad Digital Repository: http://dx.doi.org/10.5061/dryad.qp188 [39].
